# Evaluation of *Spirulina platensis* extract as natural antivirus against foot and mouth disease virus strains (A, O, SAT2)

**DOI:** 10.14202/vetworld.2015.1260-1265

**Published:** 2015-10-31

**Authors:** Hind M. Daoud, Eman M. Soliman

**Affiliations:** 1 Department of Foot and Mouth, Veterinary Serum and Vaccine Research Institute, Abbassia, Cairo, Egypt; 2 Central Laboratory for Evaluation of Veterinary Biologics, Abbassia, Cairo, Egypt

**Keywords:** antiviral, foot and mouth disease virus, *Spirulina platensis*

## Abstract

**Aim:**

This work was aimed to document the antiviral activates of *Spirulina platensis* extract against foot and mouth disease virus (FMDV) different types to evaluate its replication in Baby Hamster Kidney (BHK) cell culture and in baby mice.

**Materials and Methods:**

Cytotoxicity assay studied for *S. platensis* extract on BHK cells to determine the non-toxic dose. The non-toxic dose of Spirulina extract was mixed with each type of FMDV (A, O, SAT2). Then 10-fold dilutions from each mixture were done. FMDV titer for each type of treated FMDV was calculated to evaluate the antiviral activity of the *Spirulina* extract against FMDV. Furthermore, old baby Swiss mice were inoculated with 0.1 ml intraperitonially from the mixture of FMDV different types and different concentration of Spirulina extracts. After 48 h post inoculation, all the baby mice examined to evaluate the antiviral action of Spirulina extract.

**Results:**

The result showed that the non-toxic doses of *S. platensis* (50 ug/ml) revealed 35.7%, 28.5%, and 31% reductions in FMDV titers Type O, A, and SAT2 on BHK cells, respectively. The same non-toxic dose gave 50% of the inhibitory concentration in baby mice without cytotoxic effect.

**Conclusion:**

This study confirmed the biological activity of the ethanol extract of *S. platensis* against FMDV Types O, A, and SAT2. From the results, *S. platensis* could be useful as antiviral lead to limitation of infection among animals during outbreaks but further studies need to evaluate the *S. platensis* on experimental or natural infected farm animals to establish the effective dose side affected period of treatment of *S. platensis*.

## Introduction

Pharmaceutical drug discoveries, for past 140 years depended largely on the process of empirical screening of a large number of pure compounds. Algal organisms are a rich source of structurally novel and biologically active metabolites. Secondary or primary metabolites produced by these organisms may be potential bioactive compounds of interest in the pharmaceutical industry [1-3].

*S. platensis* is blue-green algae belong to the family of Oscillatoriaceae in the shape of a spiral coil, living both in the sea and freshwater [4,5]. It contains various essential nutrients. It contains one of the highest protein content of 70% where 18, out of 22, essential amino acids are available. It is popular vegetarian source of complete protein [6]. Genus *S. platensis* which have applications in healthy foods, animal feed, therapeutics, and diagnostics [7-10]. Spirulina has been used as food and nutritional supplements since a long time. It is generally a rich source of protein, vitamins, essential amino acids, minerals, essential fatty acids such as ã-linolenic acid and sulfolipid [11].

Several algal species contain natural bioactive compounds that act as potent antimicrobial agents Spirulina, has some valuable antiviral and antioxidant compounds [12].

*S. platensis* used frequently as a dietary supplement had been found to exhibit many immune-stimulating and antiviral activities. It had been found to activate macrophages, NK cells, T-cells, B-cells, and to stimulate the production of interferon gamma and other cytokines. Natural substances isolated from *S. platensis* had been found to be potent inhibitors against several enveloped viruses by blocking viral absorption penetration and some replication stages of progeny viruses after penetration into cells [13].

Spirulina has been shown to have important antiviral activity, when administered at a low concentration it results in reduced viral replication whereas, at higher concentrations it blocks replication. A water soluble extract of Spirulina has been shown to inhibit viral cell-penetration and replication of the herpes simplex virus Type 1 (HSV-1) in cultured HeLa cells in a dose-dependent manner. The Spirulina extract inhibits viral protein synthesis without suppressing host cell functions. The antiviral activity is attributed to Ca-Sp, which has been shown to inhibit replication of many viruses by inhibition of viral penetration into target cells without host toxicity [14-17].

Foot and mouth disease (FMD) is a highly infectious disease of cattle, sheep, goats, pigs, and also wild animals. FMD virus (FMDV) is the etiological agent of disease that can affect cloven-hoofed livestock; it causes an acute disease characterized by fever, lameness and vesicular lesions on the feet, tongue snout, and teats, with a high morbidity and low mortality [18]. The causative FMDV is antigenically diverse having seven distinct serotypes and many variants within them. The virus exists as seven distinct serotypes. Vaccination or recovery from infection, with one serotype does not protect against infection from other serotypes [19].

The most effective FMD vaccines consisted of chemically inactivated FMDV and can only offer complete protection after 7 days of vaccination because of the time needed to trigger an immune response [20]. It has been proposed that a combination of vaccine and antiviral agents can be more efficacious strategy to treat FMD-infected animals; however, there are currently no approved anti-FMDV drugs for the treatment or prevention of FMDV [21]. There are several studies for testing the antiviral effect of synthetic compounds, and natural compounds were done against FMD [20,22-26].

This work was aimed to document the antiviral activates of *S. platensis* extract against FMDV different types to evaluate its replication in Baby Hamster Kidney (BHK) cell culture and in baby mice.

## Materials and Methods

### Ethical approval

Ethical approval was not necessary as animals were not used in the study at any stage.

### Algal source

The algal materials were grown in the Phycology Unit, Botany Department, Faculty of Science, Tanta University, Egypt.

### Growth conditions

The basal medium was adopted by Zarrouk [27], Raoof *et al*. [28]. The algal was cultivated on liquid Zarrouk medium. *S. platensis* was cultured in 3 L flasks containing 2 L Zarrouk medium (pH 9). The cultures were gassed with 0.3% CO in air, and the algae were cultivated at 25±3°C for 15 days. The cultivated flasks were illuminated 24 h with continuous cool white fluorescent lamps at 400 W (equal 477 Lx).

### Growth measurements and harvesting

The growth rate of *S. platensis* was monitoring every 3 days through cultivation period by determining the dry weight and optical density at 670 nm The cells were harvested at the stationary phase, by centrifugation at 10,000 g (4°C) for 15 min, and the cells masses were stored at −20°C until use [29].

### Extraction of *S. platensis*

After collection, *S. platensis* samples were washed with freshwater several times to remove salts and debris and air dried. 500 g of the powdered samples were macerated and mixed with 2000 ml of 95% ethanol until such time all materials were submerged and allowed to stand for 24 h with occasional shaking in a dark condition and After 24 h, it was filtered. The crude residue was soaked again in a fresh 95% ethanol for another 48 h. The filtrate from the first and second soaking was then mixed together. The combined filtrates were concentrated using rotary evaporator at 50°C. The resulting concentrated extract was further concentrated by the use of a vacuum oven to remove residual solvent [30].

### Cell culture

The most susceptible cells for FMDV multiplication were BHK cells (BHK 21 clone 13). These cells were supplied by the Animal Virus Institute, Pirbright, UK. They were propagated at FMD Department, Veterinary Serum and Vaccine Research Institute, Abbassia, Cairo using minimum essential medium with Earl’s salts and 8-10% sterile new born calf serum according to the technique described by Macpherson and Stocher [31].

### FMDV

The used viruses in this study were the locally isolated FMDV types O/Panasia, A/Iran 05, and SAT2/2012 which were isolated during outbreaks in Egypt and confirmed by the World Reference Laboratory for FMD (WRL) Pirbright Institute, UK as O/Panasia, A/Iran 05, and SAT2/2012. Viruses were used for the production of the trivalent FMD vaccine.

### Cytotoxicity test

The cytotoxicity of *S. platensis* was evaluated according to Walum *et al*. [32], Simões *et al*. [33] in which the sample (100 mg) was dissolved in 1 ml of ethanol with adding 24 ul of ×100 of antibiotic then two-fold dilutions were done. 100 ul of each dilution were inoculated in BHK cell line previously cultured in 96 well tissue cultures plates to estimate the non-toxic dose of the tested sample. The assay was done using cell morphology evaluation by inverted light microscope.

### Cell morphology evaluation by inverted light microscope

After 24 h of incubation of BHK plates at 37°C, Where the cell monolayers were confluent, the medium was removed from each well and replaced an equal volume of (100 ul) growth media alone (cell controls) and with 100 ul of two-fold dilution of tested sample. The plates were incubated at 37°C and examined 2 days after by inverted light microscope. Cell morphology was observed daily microscopically detectable morphological alternations, such as loss of confluence, cell rounding and shrinking, and cytoplasm granulation and vacuolization. Morphological changes were scored [33].

### Antiviral assays of *S. platensis* against FMDV

#### Using BHK cells cultures

It was done according to Boseila [25] and El-Baz *et al*. [34]. Briefly, non-toxic dilution of Spirulina extracts (100 μl) was mixed with 100 μl of each type of FMDV solution (A, O, SAT2). The mixture was incubated for 30 min at 37°C, then 10-fold dilutions from each mixture were done (100 μl from the mixture add to 900 μl media…i.e.) 50 μl from each dilution inoculated in tissue culture plates BHK cells in 96 multi well-plates. The cultures were then incubated at 37°C. After 24 h, the plates were examined for viral cytopathic effect (CPE) under an inverted light microscope. The presence of 100% CPE in virus infectivity considers control. FMDV titer for each type of FMDV was calculated to evaluate the antiviral activity of the Spirulina extract against FMDV different types.

#### Unweaned baby mice

According to [23-35] 2-3 day old baby Swiss mice were inoculated with 0.1 ml intraperitonially from the mixture of FMDV different types (10^7^ ID_50_/ml) and different concentration of Spirulina extracts (six mice for each dilution). Besides that, a group of baby mice inoculated with FMDV only as a control. After 48 h post inoculation, all the baby mice examined to evaluate the antiviral action of Spirulina extract against FMDV three types.

Paralysis at hind limps of baby mice or death of infected mice means that these mice infected with FMDV death after 24 h is non-specific.

## Results

These results showed that there was a direct relationship between increasing concentration of the Spirulina extract and the cytotoxicity of the treated cells, until reaching 50 μg/ml, there was no cytotoxicity found in the treated cells. There was a clear reduction in the FMD virus titer of different strains was achieved by incubating the virus with non toxic dose of *S. platensis* extract at concentration of (50 μg/ml) compared with the non treated virus (virus control).

The result also showed the inhibitory concentration 50% (IC_50_) of *S. platensis* which give 50% mortality of infected baby mice with FMDV.

## Discussion

The current situation in the use of antiviral drugs in veterinary medicine is characterized by a novel and optimistic approach. The use of antiviral drugs in livestock animals is investigated for the treatment or control of disease on a large scale (mass treatment), whereas in companion animals an individual approach is favored. The inactivated vaccines of FMDV are not complete success to control the disease [36]. Since the FMDV is RNA virus which it has continuous antigenic drift [37]. The virucidal drugs have been studied for decreasing the outbreaks, and to reduce the number of infected animals during outbreaks. This paper studies the antiviral activity of ethanol extract of *S. platensis* against FMDV types (A, O, SAT2). Prior to evaluating antiviral activates of *S. platensis* against FMDV the cytotoxicity of the extracts on the host cells (BHK cells) was studied. The natural extract (*S. platensis*) should be active against the virus without inducing significant toxicity on the host cell. Therefore, the maximum concentration of the *S. platensis* at which there is no visible toxicity to the cell was determined. In the study of Kasetsart [38], the antiviral activity of 42-plant crude-extracts against the FMDV Type O on BHK cells was studied. The concentration of crude-extracts (0.2 g/ml) was two-fold diluted with Dulbecco’s modified Eagle’s medium adding 5% fetal bovine serum for cytotoxicity testing on BHK-21.

Regarding the results demonstrated in Table-1, it was showed clearly that the cytotoxicity of the BHK cells was directly proportional to the concentration of the Spirulina extract where increased concentration of the extract was accompanied by changes in cell morphology. At all concentration above (100 μg/ml) showed 100% toxicity, the cells became rounded and nuclei were more prominent, and the cells were found to float in the medium. 50% cytotoxicity appear at this concentration cells were found to be similar to apoptotic cells, However, at a concentration of 50 μg/ml the cells appear normal, and there was no cytotoxicity. These results agree with Abdo *et al*. [39] who recorded the non-toxic concentration of methanol and water extracts of *S. platensis*, *Anabaena sphaerica*, *Chroococcus turgidus*, *Oscillatoria limnetica*, and *Cosmarium* leaves in Hep-2 cell line was 2 mg/ml while in the study of El-Baz *et al*. [34]. They found that the non-toxic dose of Spirulina extract was ranged from 1.6 to 1.9 mg/ml which means no big difference between the different cell lines including buffalo green monkey kidney cells, Hep-2 derived from human, epidermoid larynx carcinoma, MA104, derived from embryonic rhesus monkey kidney tissue, and CaCo-2 heterogeneous human.

**Table-1:** Cytotoxicity of *S. platensis* on BHK cells.

Dilution of *S. platensis* extract	Concentration of *S. platensis*	Cytotoxicity % on BHK cells
1/2	50 mg	100
1/4	25 mg	100
1/8	12.5 mg	100
1/16	6.25 mg	100
1/32	3.13 mg	100
1/64	1.6 mg	100
1/128	0.8 mg (800 ug)	100
1/256	0.4 mg (400 ug)	100
1/512	0.2 mg (200 ug)	100
1/1024	0.1 mg (100 ug)	50
1/2048	0.05 mg (50 ug)	0

BHK=Baby Hamster Kidney, *S. platensis=Spirulina platensis*

Concerning the results in Table-2, it was noticed that the non-toxic dose of *S. platensis* (50 μg/ml) revealed 35%, 28.5%, and 31% reduction in infectious titer of FMDV Types A, O, and SAT2, respectively. Where the mean of virus titer was 10^7^ tissue culture infectious dose (TCID) 50/ml before treatment the virus with Spirulina extract became 10^4.5^, 10^5^, 10^4.8^ TCID 50/ml for FMDV (O, A, and SAT2), respectively, after treatment our results come to agree with those obtained by Walum *et al*. [32] who recorded that 53.3%, 66.7%, 76.7%, 56.7%, and 50% reductions *in vitro* for infectious units of adenovirus Type 7, coxsackievirus B4, astrovirus Type 1, rotavirus Wa strain, and adenovirus Type 40, respectively, treated with Spirulina extract also the result come to agree with Corona *et al*. [40] who mentioned that hot water extract of Spirulina maxima inhibited the infection for adenovirus Type 3 with a percentage <20%, with an IC_50_ 5.2 mg/ml. The mode of action of *S. platensis* is inhibits viral protein synthesis without suppressing host cell functions.

**Table-2:** Antiviral effect of non-toxic dose of *S. platensis* on FMDV titers expressed as log_10_ TCID_50_/ml.

FMDV strain	Number of virus samples	Initial titer of tested virus samples*	Final titer of tested virus samples*	Mean of final titer of tested virus samples	Mean % of reduction**
O/Panasia	S1	6	3.5	4.5	35.7
	S2	7	4		
	S3	8	6		
A/Iran 05	S4	6	3.5	5	28.5
	S5	7	5		
	S6	8	6.5		
SAT2/2012	S7	6	4	4.8	31
	S8	7	4.5		
	S9	8	6		
Virus control	S10	7	7	7	-

* Virus titer values are expressed as log_10_ TCID_50_/ml,

** Percentage of reduction of virus titer. TCID=Tissue culture infectious dose, FMDV=Foot and mouth disease virus, *S. platensis=Spirulina platensis*

On another hand the antiviral activity could be due to highly polar compounds present in ethanol extract of *S. platensis* in agreement with El-Baz *et al*. [34] and Abdo *et al*. [39] who explained the highly polar compounds in the methanol and ethanol extract of *S. platensis* on adenovirus Type 40, while Corona *et al*. [40] reported that Spirulina maxima methanol extract did not have a virucidal effect on herpes virus, inhabitation of herpes virus infection was explained by blocking the adsorption and penetration events of the 7^th^ viral replication cycle.

Regarded the result in Table-3, the inhibitory concentration of *S. platensis* 50% in baby mice was 20 μg/ml for FMDV Types O and SAT2 and 50 ug/ml for Type A. This result come to agree with Richards *et al*. [35] who showed that the prophylactic administration of the marine algae extracts was effective in reducing final mortality or prolonging the mean day of death of inoculated mice by the intra peritoneal, intra cerebral, or intranasal routes with HSV-2, beside that Hayashi *et al*. [41] found that Spirulina enhances the immune response, particularly the primary response, by stimulating macrophage functions, phagocytosis, and interleukin-1 production in baby mice. Moreover, Neekhra *et al*. [42] concluded that *S. platensis* have potent antinociceptive and anxiolytic activity in Swiss albino mice.

**Table-3:** The effect of different concentrations of *S. platensis* against different types of FMDV in baby mice.

*S. platensis* concentration	Number of baby mice	*S. platensis* concentration					

		FMDV Type (O)		FMDV Type (A)		FMDV Type (SAT2)	
		
		Number of dead mice	IC (%)	Number of dead mice	IC (%)	Number of dead mice	IC (%)
5 ug/ml	6	6	0	6	0	6	0
10 ug/ml	6	4	33	6	0	5	17
20 ug/ml	6	3	50*	5	17	3	50
50 ug/ml	6	2	67	3	50	2	67
100 ug/ml	6	1	83	2	67	2	67
200 ug/ml	6	0	100	1	83	1	83
Virus control	6	6	0	6	0	6	0

* Give 50% mortality of infected baby mice. IC=Inhibitory concentration of *S. platensis*. FMDV=Foot and mouth disease virus, *S. platensis=Spirulina platensis*

## Conclusion

This study confirmed the biological activity of the ethanol extract of *S. platensis* against FMDV Types O, A, and SAT2. From the results, *S. platensis* is could be useful as antiviral lead to limitation of infection among animals during outbreaks but further studies need to evaluate the *S. platensis* on experimental or naturally infected farm animals to establish the effective dose side affected period of treatment of *S. platensis*.

## Authors’ Contributions

HMD: Designed the study. HMD and EMS: Preparation and extraction to *S. platensis*, Preparation of BHK, determine the non toxic dose of *S. platensis* against BHK, study the effect of *S. platensis* as antivirus on FMD serotype O, A and SAT2 on BHK cell and on baby mice, drafted and revised the manuscript. Both authors read and approved the final manuscript.
